# Supplementary material for: Mitochondrial CoQ deficiency is a common driver of mitochondrial oxidants and insulin resistance
Source: eLife. 2018 Feb 6;7:e32111. doi: 10.7554/eLife.32111 (PMC5800848; doi:10.7554/eLife.32111)
Supplement: Transparent reporting form [file elife-32111-transrepform.docx]

***eLife’s* transparent reporting form**

We encourage authors to provide detailed information *within their submission* to facilitate the interpretation and replication of experiments. Authors can upload supporting documentation to indicate the use of appropriate reporting guidelines for health-related research (see [EQUATOR Network](http://www.equator-network.org/%20)), life science research (see the [BioSharing Information Resource](https://biosharing.org/)), or the [ARRIVE guidelines](http://www.plosbiology.org/article/info:doi/10.1371/journal.pbio.1000412) for reporting work involving animal research. Where applicable, authors should refer to any relevant reporting standards documents in this form.

If you have any questions, please consult our Journal Policies and/or contact us: [editorial@elifesciences.org](mailto:editorial@elifesciences.org).

**Sample-size estimation**

- You should state whether an appropriate sample size was computed when the study was being designed
- You should state the statistical method of sample size computation and any required assumptions
- If no explicit power analysis was used, you should describe how you decided what sample (replicate) size (number) to use

Please outline where this information can be found within the submission (e.g., sections or figure legends), or explain why this information doesn’t apply to your submission:

Sample size calculations were not performed. Replicate and sample sizes were determined based on experience with each specific assay. The number of replicates and mice per treatment group in each experiment is described in figure legends.

**Replicates**

- You should report how often each experiment was performed
- You should include a definition of biological versus technical replication
- The data obtained should be provided and sufficient information should be provided to indicate the number of independent biological and/or technical replicates
- If you encountered any outliers, you should describe how these were handled
- Criteria for exclusion/inclusion of data should be clearly stated
- High-throughput sequence data should be uploaded before submission, with a private link for reviewers provided (these are available from both GEO and ArrayExpress)

Please outline where this information can be found within the submission (e.g., sections or figure legends), or explain why this information doesn’t apply to your submission:

All information on biological replicates / independent experiments reported in figure legends. An independent experimental/biological experiment was defined as a separate mouse or separate in vitro experiment.

**Deposited data**

Mouse and cell line mass spectrometry data;

ProteomeXchange Consortium -PXD005128.

Reviewer account details:

Username: reviewer95675@ebi.ac.uk

Password: MxiRm9dT

Human mass spectrometry data;

ProteomeXchange Consortium - PXD006891

Reviewer account details:

Username: reviewer37567@ebi.ac.uk

Password: DDn6Tdv9

Mouse and cell line microarray data; https://www.ncbi.nlm.nih.gov/geo/query/acc.cgi?acc= GSE87853

The following secure token has been created to allow review of record GSE87853 while it remains in private status:

Ctgjiwokbpkrngp

https://www.ncbi.nlm.nih.gov/geo/query/acc.cgi?acc= GSE87854

The following secure token has been created to allow review of record GSE87854 while it remains in private status:

kxujskgqlbuvpuv

**Statistical reporting**

- Statistical analysis methods should be described and justified
- Raw data should be presented in figures whenever informative to do so (typically when N per group is less than 10)
- For each experiment, you should identify the statistical tests used, exact values of N, definitions of center, methods of multiple test correction, and dispersion and precision measures (e.g., mean, median, SD, SEM, confidence intervals; and, for the major substantive results, a measure of effect size (e.g., Pearson's r, Cohen's d)
- Report exact p-values wherever possible alongside the summary statistics and 95% confidence intervals. These should be reported for all key questions and not only when the p-value is less than 0.05.

Please outline where this information can be found within the submission (e.g., sections or figure legends), or explain why this information doesn’t apply to your submission:

Information provided within each figure legend, and in the Materials and Methods section under the headings “Differential transcript and protein analysis”, “Pathway enrichment analysis”, “Pathway integrative analysis and phenotype association” and “Statistical Analyses”.

(For large datasets, or papers with a very large number of statistical tests, you may upload a single table file with tests, Ns, etc., with reference to sections in the manuscript.)

**Group allocation**

- Indicate how samples were allocated into experimental groups (in the case of clinical studies, please specify allocation to treatment method); if randomization was used, please also state if restricted randomization was applied
- Indicate if masking was used during group allocation, data collection and/or data analysis

Please outline where this information can be found within the submission (e.g., sections or figure legends), or explain why this information doesn’t apply to your submission:

For mouse studies, cages were randomly assigned to chow or high fat high sucrose diet. For saline/CoQ administration, mice within cages were randomly assigned to a treatment group.

For human studies, subjects were designated into “insulin sensitive” or “insulin resistant” subgroups based on clinical measures during a hyperinsulinaemic-euglycaemic clamp as described in the text.

In experiments determining CoQ concentrations, investigators were blinded to tissue origin / sample treatment group.

**Additional data files (“source data”)**

- We encourage you to upload relevant additional data files, such as numerical data that are represented as a graph in a figure, or as a summary table
- Where provided, these should be in the most useful format, and they can be uploaded as “Source data” files linked to a main figure or table
- Include model definition files including the full list of parameters used
- Include code used for data analysis (e.g., R, MatLab)
- Avoid stating that data files are “available upon request”

Please indicate the figures or tables for which source data files have been provided:

All mRNA and proteomic analyses used for this manuscript are provided as supplemental tables (Tables S1-S3).
